# Supplementary material for: A High Proportion of Malaria Vector Biting and Resting Indoors despite Extensive LLIN Coverage in Côte d’Ivoire
Source: Insects. 2023 Sep 12;14(9):758. doi: 10.3390/insects14090758 (PMC10532360; doi:10.3390/insects14090758)
Supplement: Supplementary file 1 [file insects-14-00758-s001.zip › insects-2518000-supplementary.pdf]

SM Supplementary Material

Table S1: Number of sampled houses with PSC and WT in the two villages during each months of collections

Village	Sampling Methods	Months of collections			
		December	March	May	October
Coastal	WT	20	20	20	20
	PSC	11	11	11	15
Inland	WT	20	20	20	20
	PSC	20	20	20	18

Table S2: Summary of female *Anopheles* mosquitoes collected from PSC and WT in coastal and inland villages in Côte d'Ivoire

Study sites and Anopheles	PSC	WT
Coastal		
An. gambiae s.l.	151	118
An. nili	1	3
An. coustani	1	0
An salbaii	1	0
An funestus	0	0
Inland		
An. gambiae s.l.	162	146
An. nili	0	0
An salbaii	0	0
An. coustani	0	0
An funestus	43	17

Table S3: Result of LM-1 of *An. gambiae* s.l. female m/p/n in the coastal and inland village in with PSC and WT sampling methods. Inland village and WT sampling method collections taken as reference level.

Variables	Estimates	Std. Errore	t-values	Pr (>|t|)
Intercept	0,503	0,241	2,081	0,036*
PSC	0,578	0,334	1,679	0,094
Coastal	0,263	0,342	0,770	0,442
PSC*Coastal	-0,098	0,523	-0,188	0,851

Table S4: Result of GLM-2 of *An. gambiae* s.l. female Plasmodium infection rate in the coastal and inland village in with PSC and WT sampling methods. Inland village and WT sampling method collections taken as reference level.

Variables	Estimates	Std. Errore	t-values	Pr (>|t|)
Intercept	-2,951	0,387	-7,614	2,66E-14***
PSC	2,335	0,442	5,28	1,29E-07***
Coastal	0,025	0,57	0,044	0,965
PSC*Coastal	-0,78	0,663	-1,176	0,241

Table S5: Blood meal origins of *An. gambiae* s.l. collected by PSC and WT in Coastal and Inland villages in Cote D'Ivoire.

Village	Blood-meal origins	PSC	WT
Coastal	Number tested	42	8
	Human	97,6% (41)	87,5% (7)
	Bovine/Goat	0	12,5 %(1)
	Human+Dog	2,4 (1)	0
	HBI	100%	87,50%
Inland	Number tested	39	2
	Human	100% (39)	100% (2)
	Bovine/Goat	0	0
	Human+Dog	0	0
	HBI	100%	100%

Table S6: Result of GLMM-3 of blood-fed/ empty *An. gambiae* s.l. female coastal and inland village with PSC and WT sampling methods. WT sampling method collections taken as reference level.

Village	Variables	Estimates	Std. Errore	t-values	Pr (>|t|)
Coastal	Intercept	-1.140	0.583	-1.953	0.051
	PSC	3.073	0.849	3.620	0.0002
Inland	Intercept	-2.851	0.617	-4.616	3.90e-06
	PSC	3.774	0.788	4.789	1.67e-06

Table S7: Observed mean proportion of people sleeping under LLIN aggregate for month of collecitons in each sampling house and number of *An. gambiae* collected blood fed and empty in the coastal and inland village.

Village	House Number	Month	Mean people sleeping under LLIN (%)	Number of people sleeping in the house	Number of people sleeping in the house under LLINs	Number of *An. gambiae* blood fed	Number of *An. gambiae* empty
Coastal	1	December	100	4	4	0	0
		March	100	4	4	0	0
		May	100	4	4	1	9
		October	100	12	12	0	0
	2	December	100	8	8	0	0
		March	100	8	8	0	0
		May	40	5	2	1	0
		October	0	12	0	0	0
	3	December	100	8	8	0	0
		March	100	8	8	0	0
		May	100	8	8	8	32
		October	100	8	8	0	0
	4	December	0	12	0	3	8
		March	0	12	0	0	0
		May	50	12	6	22	14
		October	0	4	0	0	0
	5	December	100	4	4	1	8
		March	100	4	4	0	2
		May	100	10	10	5	4
		October	100	4	4	0	0
	6	December	100	2	2	1	0
		May	0	1	0	3	1
		October	0	3	0	0	0
	7	December	50	2	1	0	0
	8	December	100	2	2	0	0
	9	December	100	1	1	3	0
	10	December	0	2	0	2	0
	11	December	0	4	0	0	0
	12	December	0	1	0	3	0
	13	December	0	3	0	0	0
		May	100	1	1	2	0
		October	100	1	1	0	0
	14	December	100	3	3	0	1
		May	0	4	0	12	2
		October	100	1	1	1	0
	15	December	100	3	3	8	1
		May	100	4	4	0	0
	16	December	0	2	0	0	0
	17	March	100	2	2	0	0
		May	100	1	1	1	2

	18	March	0	1	0	2	0
	19	March	66	3	2	4	0
	20	March	100	2	2	0	0
	21	March	66	3	2	3	0
		May	100	4	4	0	0
		October	100	2	2	0	0
	22	March	100	2	2	1	3
	23	March	100	2	2	0	0
	24	March	100	2	2	6	0
	25	March	0	4	0	6	4
	26	March	0	4	0	21	3
	27	March	50	4	2	9	0
	28	May	33	6	2	6	10
		October	20	10	2	5	1
	29	May	100	3	3	4	1
		October	100	1	1	0	0
	30	May	100	1	1	0	0
	31	May	0	3	0	0	0
	32	May	100	2	2	5	7
	33	October	100	3	3	0	1
	34	October	100	1	1	1	0
	35	October	100	1	1	0	0
	36	October	100	2	2	0	0
	37	October	100	1	1	0	0
	38	October	100	1	1	0	0
	39	October	100	2	2	2	0
	40	October	100	1	1	0	0
inland	1	December	100	16	16	1	2
		March	100	16	16	0	0
		May	100	16	16	1	0
		October	100	16	16	1	39
	2	December	100	12	12	0	0
		March	100	12	12	0	0
		May	100	12	12	0	0
		October	100	12	12	0	0
	3	December	100	16	16	0	0
		March	100	16	16	0	0
		May	100	16	16	0	0
		October	100	16	16	0	14
	4	December	100	28	28	0	0
		March	100	4	4	0	1
		May	100	4	4	1	0
		October	100	4	4	3	10
	5	December	100	16	16	0	0
		March	100	16	16	0	0
		May	100	28	28	2	3
		October	100	28	28	0	63

6	December	100	2	2	2	0
	March	100	2	2	0	0
	May	100	2	2	0	0
	October	100	2	2	0	0
7	December	100	1	1	0	0
	March	0	3	0	0	0
	May	0	3	0	0	0
	October	0	3	0	8	0
8	December	100	2	2	2	1
	March	0	1	0	0	0
	May	0	1	0	0	0
	October	0	1	0	27	0
9	December	0	1	0	0	0
	March	100	1	1	0	0
	May	100	1	1	0	1
	October	100	1	1	3	1
10	December	0	3	0	0	0
	March	100	2	2	0	0
	May	100	2	2	0	0
	October	100	2	2	0	0
11	December	100	4	4	0	1
	March	100	4	4	0	0
	May	100	4	4	0	0
	October	100	4	4	1	0
12	December	0	6	0	4	1
	March	0	6	0	0	0
	May	0	6	0	0	1
	October	0	6	0	1	0
13	December	0	4	0	1	4
	March	0	4	0	0	0
	May	0	4	0	0	0
	October	0	4	0	1	0
14	December	100	5	5	0	0
	March	100	5	5	0	0
	May	100	5	5	1	0
	October	100	5	5	0	4
15	December	100	4	4	0	0
	March	100	4	4	1	0
	May	100	4	4	1	1
	October	100	4	4	1	0
16	December	100	2	2	2	5
	March	100	2	2	0	0
	May	100	2	2	2	0
	October	100	2	2	4	0
17	December	100	2	2	0	2
	March	100	2	2	0	0
	May	100	2	2	1	0
	October	100	2	2	1	2

18	December	100	7	7	1	2
	March	100	7	7	0	0
	May	100	7	7	0	2
	October	100	7	7	0	0
19	December	100	3	3	2	3
	March	100	3	3	0	0
	May	100	3	3	0	0
	October	100	3	3	0	0
20	December	100	2	2	0	0
	March	100	4	4	0	0
	May	100	4	4	0	0
21	December	100	3	3	0	0
	March	100	2	2	0	0
	May	100	2	2	7	3
	October	100	2	2	9	5
22	December	100	5	5	1	0
	March	100	2	2	2	0
	May	100	2	2	1	0
	October	100	2	2	1	1
23	December	100	1	1	0	0
	March	100	1	1	1	0
	May	100	1	1	1	0
	October	100	1	1	0	0
24	December	100	2	2	3	2
	March	100	5	5	0	0
	May	100	5	5	2	0
25	December	100	2	2	1	0
	March	100	3	3	2	5
	May	100	3	3	2	2
	October	100	3	3	0	0

Table S8: Result of GLMM-4 of blood fed/ empty *An. gambiae* s.l. female in coastal and inland village with PSC and WT sampling methods as function of usage of LLINs.

<table>
<tr><th>Variables</th><th>Estimate</th><th>Std.Errore</th><th>z value</th><th>Pr(>|z|)</th></tr>
<tr><td>Intercept</td><td>1.945</td><td>0.436</td><td>4.459</td><td><0.001</td></tr>
<tr><td>Yes LLINs</td><td>-2.906</td><td>0.459</td><td>-6.325</td><td><0.001</td></tr>
<tr><td>Coastal Village</td><td>-1.271</td><td>0.496</td><td>-2.562</td><td>0.01</td></tr>
<tr><td>Coastal Village*Yes LLINs</td><td>2.177</td><td>0.542</td><td>4.015</td><td><0.001</td></tr>
</table>

Table S9: Result of GLMM-4 of blood fed/ empty *An. gambiae* s.l. female in coastal and inland village only with PSC samples.

<table>
<tr><th>Variables</th><th>Estimate</th><th>Std.Errore</th><th>z value</th><th>Pr(>|z|)</th></tr>
</table>

**Intercept**	1.945	0.436	4.459	<0.001
**Yes LLINs**	-1.646	0.480	-3.426	<0.001
**Coastal Village**	-0.356	0.557	-0.640	0.522
**Coastal Village*Yes LLINs**	0.842	0.665	1.263	0.206
